# Does moderate or severe nonspecific knee injury affect radiographic osteoarthritis incidence and progression?

**DOI:** 10.1186/1471-2474-14-309

**Published:** 2013-10-28

**Authors:** Eric C Sayre, Joel Singer, Anona Thorne, Hubert Wong, Jacek A Kopec, John M Esdaile, Ali Guermazi, Savvas Nicolaou, Jolanda Cibere

**Affiliations:** 1 Arthritis Research Centre of Canada, 5591 No. 3 Rd, Richmond, BC, V6X 2C7, Canada; 2 School of Population & Public Health, University of British Columbia (UBC), Vancouver, Canada; 3 Medicine, UBC, Vancouver, Canada; 4 Medicine, University of Calgary, Calgary, Canada; 5 Radiology, Boston University School of Medicine, Boston, USA; 6 Radiology, UBC, Vancouver, Canada

## Abstract

**Background:**

Knee injuries can lead to radiographic osteoarthritis (ROA). Injuries may be “specific” (SI) including ligament or meniscal tears or patellar trauma, or “nonspecific” (NSI). Our objective is to understand the effect of knee NSI on ROA incidence and progression.

**Methods:**

163 people (sample-weighted for population representativeness) aged 40+ with history of knee pain had radiographs assessed on Kellgren Lawrence (KL) grade (0/1 collapsed) at baseline and follow-up (median 3.2 years apart). Progression was an increase in KL score. SIs and NSIs were labeled “severe” (walking aid for ≥1 week) or “moderate”. One model treated SI and NSI as dichotomous (yes/no), and another as trichotomous (none/moderate/severe). Models were adjusted for age, sex, BMI, KL grade and follow-up time.

**Results:**

SI/NSI history was none, moderate (7.8/24.4%) or severe (11.0/10.8%). Duration at baseline since SI/NSI ranged from <1 year to several decades (SI/NSI mean 4.6/6.5 years). SI was significantly associated with ROA incidence and progression (odds ratio (OR) = 2.90; 95% CI = 1.04, 8.09), but NSI showed no significant effect (OR = 1.36; 95% CI = 0.61, 3.02). In the trichotomous model, severe SI was significant (OR = 4.35, 95% CI = 1.26, 15.02), while moderate SI was not (OR = 1.51, 95% CI = 0.33, 6.84). NSI showed no effect: moderate OR = 1.51, 95% CI = 0.61, 3.74; severe OR = 0.90, 95% CI = 0.24, 3.40. This study had 80% power to detect an NSI OR of 2.9.

**Conclusion:**

We find no evidence that history of NSI affects knee ROA incidence and progression in a population with knee pain, adjusting for SI, age, sex, BMI, KL grade and follow-up time.

## Background

Among US adults, nearly 27 million had clinical osteoarthritis in 2008 (up from 21 million in 1995) [1]. Being strongly related to age and body mass index (BMI), with the increasing average age and adiposity of populations, osteoarthritis (OA) presents an increasing burden [2-5]. Kellgren Lawrence (KL) grade, an integer index ranging from 0 to 4, is a standard radiographic measurement of joint deterioration used in diagnosing OA [6]. Radiographic OA (ROA) is simply defined as a KL grade of 2 or higher. In this paper we study incidence and progression of ROA, defined as any increase in KL grade (with grades 0/1 collapsed).

There is a large body of literature suggesting that knee injury leads to an increased risk of OA (mostly incident or prevalent OA) [7-17]. However, while the general topic of knee injury affecting OA has been studied fairly extensively, it has either been limited to the study of certain specific injuries (SIs) that have been widely identified as posing elevated risks for OA (torn ligament, torn meniscus, injured/dislocated patella), or used a definition that included these injuries. The present study seeks to elucidate the effects (if any) of injuries that do not fall within those specific categories. We term this “nonspecific injury” (NSI). Similar to the specific injuries listed above, NSI can arise during physical exercise, or activities of daily living, and may be mild or severe. We define both SI and NSI as either “severe” (requiring a walking aid for at least one week), or else “moderate”. In comparison to the topic of SI versus OA, there is a dearth of literature on the area of NSI (as we have defined it) and OA, yet the long-term effect of non-specific knee injuries is important to understand, as many knee injuries are nonspecific. The purpose of this study is to better understand the effect of moderate and severe knee NSI, defined as a knee injury other than torn ligament, torn meniscus, or injured/dislocated patella, on the long term rate of ROA incidence/progression.

## Methods

### Ethics approval

The study was approved by the Research Ethics Board of the University of British Columbia. All participants gave written informed consent at the time of recruitment.

### Data collection

Source data came from a longitudinal study conducted in Vancouver, Canada [18], a population-based cohort of individuals aged 40 to 79 with knee pain “on most days of the month at any time in the past and any pain in the past 12 months.” This cohort enrolled 255 individuals, stratified by age decade and sex in roughly equal group sizes, with baseline visits between 2002 and 2005. The sample was collected as follows. A random list of households was obtained from the telephone directory listings, which included address and telephone information. Invitation letters were mailed to randomly selected households. This was followed by standardized telephone screening for preliminary eligibility, followed by in-person detailed eligibility screening. Once predetermined numbers were reached in a given age-sex group, data collection stopped within that group. The first time this happened, the sample collected to that point provided the estimated population proportional distribution of age and sex in those with knee pain. This distribution was used to develop a sample weight (see below). Collection continued until sufficient numbers were reached in each group [19].

A total of 163 patients completed follow-up over 2.5 to 5.6 years (mean = 3.3, SD = 0.5), with the remainder of the baseline sample not available for the second x-ray.[18] The study knee was the more painful knee. Radiographs were taken using fixed-flexion anteroposterior view and skyline view. X-rays were graded using the Kellgren Lawrence scale (0–4). Interrater reliability of KL grade was good with a baseline intraclass correlation coefficient of 0.79. Differences were adjudicated by consensus readings among the two readers (JC and SN). Grades 0 and 1 were collapsed, then ROA incidence and progression was defined as an increase by ≥ 1 grade. Subjects with KL grade 4 are not able to progress and hence were excluded from this analysis.

History of knee injury was obtained by self report. Subjects were asked: “Have you had any knee injuries in the past? Please list all injuries to the right and left knee (for example meniscal tear, strain, anterior cruciate injury). Be as specific as you can. Indicate when the injury occurred, whether you required walking aids for at least one week, which knee was affected and how certain you are of which knee was affected.” The response box to this question contained rows for as many as eleven injuries, with fields for type of injury (open-ended description), date of injury, walking aids required for at least one week (yes/no), which knee injured (right/left), and certainty about which knee was injured (not/somewhat/very certain). We did not review medical charts and did not obtain imaging information at the time of injury, since injuries could have occurred many years prior to the study visit. However self-reported information on all injuries was reviewed in detail by a rheumatologist (JC) at time of study visit. Subjects with non-specific injuries may or may not have had imaging at the time of injury. Regardless of imaging that they might have had, they were not aware of any specific diagnoses that would be codable as another type of specific injury. Type of injury was classified by a rheumatologist (JC) as either specific injury (ACL tear, cruciate ligament tear, LCL tear, MCL tear, (other) ligament tear, meniscal tear, patellar injury), or else was labeled nonspecific injury. SI and NSI severity were defined as severe (requiring a walking aid for at least one week) or else moderate [18]. In the case of multiple SIs, duration from the oldest was specified as the SI duration at study baseline, and similarly the maximum SI severity was treated as the SI severity. In the case of multiple NSIs, the same approach was taken. Subjects may have experienced both SI and NSI.

### Statistical methods

In order to obtain results that were population-representative, a sample weight was developed for the baseline sample as the ratio of population proportion in a given age-sex cell over the baseline sample proportion in that cell. The weight was scaled to sum to the baseline sample size (255). The longitudinal subset analyzed in the present study consisted of 163 subjects followed up from the original baseline sample [18]. A sample weight was developed for the longitudinal sample as the ratio of baseline sample proportion in a given age-sex cell over the longitudinal sample proportion in that cell, multiplied by the baseline weight. The sample weight was scaled to sum to the follow-up sample size (163). All analyses in the present study were weighted with the longitudinal sample weight.

Logistic regression was used to model ROA incidence and progression in two ways. One logistic model treated SI and NSI as dichotomous (yes/no). In another model, trichotomous variables were used for both SI and NSI (none/moderate/severe). Both models included SI and NSI together (i.e., NSI models were adjusted for SI), and were also adjusted for baseline age, sex, BMI, KL grade and follow-up time (2.5 to 5.6 years as described above). Interactions were allowed for between sex and NSI, and between sex and SI. They were dropped if non-significant at alpha = 0.05. Model fit was assessed with the Hosmer and Lemeshow goodness of fit test for logistic regression models [20]. As stated above, regression models were fit on the group with KL grade <4 at baseline (unweighted/weighted n = 147/149.6). Logistic regression does not require that SI and NSI be mutually exclusive. In fact, that they are not mutually exclusive is what allows the model to estimate the effect of each while adjusting for the other. Both were evaluated in the same model using “none” as the reference groups.

The following four sensitivity analyses were performed to assess the robustness of the results to various assumptions and limitations. First, NSI models were fit on the subgroup without history of SI, in effect a “purified” cohort. Secondly, to investigate a possible effect of time between injury and baseline radiographic assessment, we performed an analysis in which NSI and SI were treated as 5-level variables with categories “none”, “moderate recent (≤20 years)”, “moderate old (>20 years)”, “severe recent (≤20 years)”, and “severe old (>20 years)”. In a third sensitivity analysis, we added any SI or NSI to the study knee occurring between baseline and follow-up as additional positives for history of SI or NSI, and fit the models on the updated variables. The fourth sensitivity analysis was performed to investigate the possibility of different effects on ROA incidence and progression. This was done by considering results from separate incidence and progression models in combination with a cross-tabulation of baseline ROA prevalence versus trichotomous SI and NSI history.

Finally, we compared the followed up subsample of 163/255 to the subsample that was not followed up (92/255), on baseline values of specific injury, non-specific injury, KL grade, gender, age and BMI.

Analyses were performed using the statistical software SAS version 9.2.

## Results

Weighted characteristics of the full sample (N = 163.0) are described in Table 1. 54.0 % were female. At baseline, average BMI was 26.1 (range = 18.1-43.2, SD = 4.2). 39.4% had baseline ROA. By follow-up, 35.0% had progressed on the KL grade (0/1 collapsed) of their study knee. The vast majority of subjects’ study knees progressed or remained the same, with only n = 3.0 (1.8%) moving backwards on the collapsed KL scale. Those were treated as non-progressors. At baseline, 24.4% had experienced moderate knee NSI, and 10.8% severe knee NSI. 7.8% had experienced moderate knee SI, and 11.0% severe knee SI. Duration from the oldest SI to baseline radiographic assessment ranged from 1–58 years, while duration from the oldest NSI to baseline radiographic assessment ranged from 0–70 years.

**Table 1:** Sample characteristics (weighted counts; N = 163.0)

**Variable**	**n (%)**
Baseline history of knee NSI	
None	105.5 (64.7)
Moderate	39.8 (24.4)
Severe	17.7 (10.8)
Baseline history of knee SI	
None	132.4 (81.2)
Moderate	12.7 (7.8)
Severe	17.9 (11.0)
^1^Baseline KL	
0/1	98.9 (60.6)
2	34.5 (21.2)
3	16.3 (10.0)
4	13.4 (8.2)
^1^Progressors (KL increase by ≥ 1 grade at follow-up)	57.0 (35.0)
Female	88.1 (54.0)
Baseline age, mean (SD)	57.6 (10.1)
Baseline BMI, mean (SD)	26.1 (4.2)
Years of follow-up, mean (SD)	3.3 (0.6)

^1^KL 0/1 is treated as one category.

Table 2 lists the odds ratios and 95% confidence intervals from the regression models, adjusted for age, sex, baseline BMI, baseline KL grade and years of follow-up. Interactions were dropped due to non-significance at alpha = 0.05, so the odds ratios apply to both sexes. In the dichotomous model, SI had a significant association with ROA incidence and progression (OR = 2.90; 95% CI = 1.04, 8.09), but NSI did not show an association in the multivariable model (OR = 1.36; 95% CI = 0.61, 3.02). In the trichotomous model, the effects of SI were monotonic. Severe SI was significantly associated with ROA incidence and progression, while moderate SI was not: moderate SI OR = 1.51 (95% CI = 0.33, 6.84); severe SI OR = 4.35 (95% CI = 1.26, 15.02). NSI showed no effect: moderate NSI OR = 1.51 (95% CI = 0.61, 3.74); severe NSI OR = 0.90 (95% CI = 0.24, 3.40). Both models passed the goodness of fit test at alpha = .05. Fixing the weighted number of progressors and subjects with knee NSI to what we observed post-hoc, we had a post-hoc power of 80% to detect an odds ratio of 2.9 when defining NSI as a binary variable.

**Table 2:** SI/NSI models predicting ROA incidence and progression

**Variable**	**OR (95% CI)**
Dichotomous model	
Knee NSI (ref. none)	
Any	1.36 (0.61, 3.02)
Knee SI (ref. none)	
Any	2.90 (1.04, 8.09)
Trichotomous model	
Knee NSI (ref. none)	
Moderate	1.51 (0.61, 3.74)
Severe	0.90 (0.24, 3.40)
Knee SI (ref. none)	
Moderate	1.51 (0.33, 6.84)
Severe	4.35 (1.26, 15.02)

ROA = radiographic OA.

Models are adjusted for baseline age, sex, BMI, KL grade and years of follow-up.

The results of the sensitivity analyses were as follows. In the models fit on the subgroup without history of SI, odds ratios for NSI were similar to the above results, with slightly wider confidence intervals. In the dichotomous model, OR = 1.55; 95% CI = 0.63, 3.83. In the trichotomous model, moderate NSI OR = 1.79 (95% CI = 0.66, 4.85); severe NSI OR = 1.03 (95% CI = 0.23, 4.70). In the analysis accounting for time between NSI or SI and baseline radiographic assessment, results were similar in that SI was significant while NSI was not. Only old (>20 years) severe SI was significant vs. no injury, with OR = 5.47 (95% CI = 1.41, 21.27), not recent SI or any NSI. For the analysis treating SI/NSI occurring between baseline and follow-up as additional history of injury, there were no new SIs to the study knee in that time, but there were 11 new knee NSIs, two of them severe. Addition of these events did not change the conclusions and hardly changed the estimates in dichotomous or trichotomous models. Odds ratios were within 0.01 in the significant categories, and within 0.1 in the non-significant categories. In the final sensitivity analysis, in separate incidence and progression models, we observed a significant effect of SI on progression, but none on incidence. NSI had no effect in either model. In the complementary cross-tabulation comparing the baseline prevalence of ROA versus history of knee SI and knee NSI, we found that ROA prevalence increased monotonically for levels of SI; that is, ROA prevalence was 35.0%, 49.7% and 64.3% for none, moderate and severe SI respectively. However, again, no such trend was seen for levels of NSI, where ROA prevalence was 41.0%, 34.6% and 40.7% respectively. The combination of these results (prevalence rates and separate models for incidence and progression) together support combining the outcome into incidence/progression: the length of time between injury and baseline radiographic assessment has precluded separation of the outcome into incidence and progression alone because any effect of injury on incidence has already acted by the time of radiographic assessment at study baseline. In this two-pronged sensitivity analysis, an effect of SI on incidence *is* detected (as differences in ROA prevalence at study baseline), yet no evidence of an effect of NSI is seen.

Finally, there were no statistically significant differences between the followed up subsample of 163/255 vs. the subsample that was not followed up (92/255), on baseline values of specific injury, non-specific injury, KL grade, gender, age and BMI.

## Discussion

In multivariable, adjusted models, we investigated the effects of past history of knee NSI—injuries not including the “specific” injuries cruciate ligament tear, collateral ligament tear, meniscal tear or patellar injury—on the incidence and progression of ROA. In models adjusted for knee SI, baseline age, sex, BMI, KL grade and years of follow-up, we found no evidence that history of moderate or severe knee NSI (including those severe enough to require a walking aid for at least one week) affects the incidence and progression of knee ROA in a population with knee pain. We investigated this model in a dichotomous approach with SI and NSI having levels yes/no, and a trichotomous approach with levels none/moderate/severe. In addition, we performed a series of sensitivity analyses, including an analysis of the SI-free subgroup, a model accounting for time between injury and baseline radiographic assessment, a model in which injuries occurring between baseline and follow-up were counted as additional history of injury, and finally a two-pronged analysis of separate effects on ROA incidence and progression which combined results from separate incidence and progression models with results from a cross-tabulation of baseline ROA prevalence versus trichotomous SI and NSI variables. Results were consistent across all approaches: no effect of knee NSI, but a significant effect of knee SI on subsequent incidence and progression of ROA.

The adjusted effects we found for knee SI on ROA incidence and progression are consistent with the literature on this topic [7-17]. For example, in a longitudinal study with a 22-year follow-up, Toivanen et al. (2010) reported an odds ratio (OR) of 5.1 (95% confidence interval (CI) 1.4, 19.0) for permanent complaints due to past knee injury (e.g., ligament injuries) and developing (incident) symptomatic OA [7]. Øiestad et al. (2009) performed a systematic review of knee injury versus tibiofemoral OA, with a minimum 10-year follow-up [8]. They found that reported prevalence rates of OA following cruciate ligament and additional meniscal injury ranged from 21% to 48%, two to five times higher than in the overall adult population [1]. Chaudhari et al. (2008), Boyd et al. (2005) and Allen et al. (1999) examined biological models of OA development after ACL injury [9-11]. Burger et al. (2007) used a sheep model to examine cartilage changes that may lead to OA following meniscal injury [12]. In a 14-year longitudinal study, Wilder et al. (2002) reported an OR = 7.4 (95% CI = 5.9, 9.4) for the association between self-reported history of acute knee injury (fracture or severe sprain) and development of (incident) ROA [13]. In a longitudinal study with an average follow-up time of five years, Cooper et al. (2000) reported an odds ratio for the association between previous knee injury (severe, requiring a walking aid for at least one week) and incident OA to be 4.8 (95% CI = 1.0, 24.1) [14]. In a 36-year longitudinal study, Gelber et al. (2000) reported relative risks ranging from 2.95 to 5.17 (depending on time of injury) for the association between joint injury and subsequent OA [15]. In a case–control study, Lau et al. (2000) reported odds ratios of 12.1 (95% CI = 3.4, 42.5) in men and 7.6 (95% CI = 3.8, 15.2) in women, for the association between history of joint injury and subsequent OA [16]. Sillanpää et al. (2011) found that patellofemoral OA is a significant long-term risk of nonanatomic surgery following patellar dislocation [17].

Our non-finding of any effect of knee NSI has important implications, especially if it is confirmed in a future, equivalence analysis. If confirmed, the null finding means that people who suffer non-specific injuries to the knee (no apparent damage to ligaments, meniscus or patella) need not worry about a subsequent, long-term, increased risk of radiographic OA incidence/progression, even when the NSI is severe enough to require a walking aid for at least one week. This result is important, as an increasing popularity of physical exercise may lead to more knee injuries, both specific and nonspecific. In addition, those who are overweight and are asked to begin exercise programs (which ought to reduce rates of ROA) represent another growing group at risk for knee injury.

This study has certain limitations. Our null findings do not exclude the possibility that knee NSI increases the risk of ROA incidence and progression; an equivalence study would be required to draw such conclusions. However, the 95% CIs obtained, as well as adequate power suggest that the magnitude of such an increase, if any, is likely not large. Another limitation of our study is that history of SI and NSI were obtained retrospectively, by self-report. The wide range of time between injury and baseline radiographic assessment (<1 yr to several decades) is potentially important. This may have the effect of a reduction in power. Nevertheless, we found a positive result for the effect of SI. This suggests that its impact is not substantial. In addition, sensitivity analyses adjusting for time from injury to baseline radiographic assessment did not change the conclusions of this study. The length of time between injury and baseline radiographic assessment also precluded separation of the outcome into incidence and progression alone in the primary analysis, as any effect on incidence had already acted by the time of baseline radiographic assessment. However, our analysis of ROA prevalence at baseline versus history of SI and NSI, combined with sensitivity analyses with incidence and progression modeled separately, suggest that this would not have affected the null findings of this study. Another limitation is that we did not do medical chart review of past injuries, and relied instead on patient recall. Because of this, we do not have detailed information on other tissues affected such as cartilage. This is something that should be looked at prospectively for NSI in future studies. Finally, while this study is population-based, the target population is not the general population, but those with a history of knee pain who were successfully followed up. As such, we cannot be sure that the results of this study are applicable to the cross-sectional population of those without knee pain.

## Conclusions

In summary, we did not find evidence that history of moderate or severe nonspecific knee injury—injuries not including the “specific” injuries relating to cruciate ligament tear, collateral ligament tear, meniscal tear or patellar injury—(including those severe enough to require a walking aid for at least one week) affects the incidence and progression of radiographic knee OA in a population with knee pain. This result was irrespective of adjustment for specific knee injury, baseline age, sex, BMI, KL grade and follow-up time between radiographs.

## Competing interest

Ali Guermazi is President of Boston Imaging Core Lab, LLC, and consultant to Pfizer, AstraZeneca, Novartis, Merck Serono and Genzyme. No other authors have competing interests to declare.

## Authors’ contributions

All authors on this manuscript (ES, JS, AT, HW, JK, JE, AG, SN and JC) made significant contributions to the study design. JC, JE, AG and SN were involved in acquisition of data. All authors were involved in the analysis and interpretation of data, as well as drafting and revising the manuscript. All authors gave final approval of the version to be published.

## Pre-publication history

The pre-publication history for this paper can be accessed here:

http://www.biomedcentral.com/1471-2474/14/309/prepub
